# Diagnostic accuracy of deep learning vs. human raters for detecting osteoporotic vertebral compression fractures in routine CT scans

**DOI:** 10.1007/s00330-026-12393-y

**Published:** 2026-02-24

**Authors:** Evamaria O. Riedel, David Schinz, Matthias Keicher, Sebastian Rühling, Malek El Husseini, Chantal Pellegrini, Thomas Baum, Michael Dieckmeyer, Luca Malagutti, Isabel Seeger, Anna S. Walburga, Benedikt Wiestler, Nico Sollmann, Maximilian T. Löffler, Arthur Wagner, Jan S. Kirschke

**Affiliations:** 1 Department of Neuroradiology, School of Medicine and Health, Technical University of Munich, Munich, Germany; 2 Institute of Radiology, Universitätsklinikum Erlangen, Friedrich-Alexander-Universität Erlangen-Nürnberg, Erlangen, Germany; 3 Computer-Aided Medical Procedures, Technical University of Munich, Munich, Germany; 4 Department of Diagnostic, Interventional, and Pediatric Radiology, Inselspital, University of Bern, Bern, Switzerland; 5 TranslaTUM-Central Institute for Translational Cancer Research, Technical University of Munich, Munich, Germany; 6 AI for Image-Guided Diagnosis and Therapy, School of Medicine and Health, Technical University of Munich, Munich, Germany; 7 Department of Diagnostic and Interventional Radiology, University Hospital Ulm, Ulm, Germany; 8 Department of Nuclear Medicine, University Hospital Ulm, Ulm, Germany; 9 TUM-Neuroimaging Center, TUM Klinikum Rechts der Isar, Technical University of Munich, Munich, Germany; 10 Department of Diagnostic and Interventional Radiology, University Medical Center Freiburg, Freiburg, Germany; 11 Department of Neurosurgery, University Hospital, Technical University Munich School of Medicine, Munich, Germany

**Keywords:** Spine, Osteoporosis, Osteoporotic fractures, Computed tomography, Deep learning

## Abstract

**Objectives:**

Vertebral fractures are severe complications of osteoporosis but are frequently missed on computed tomography (CT). Differentiating true fractures from non-osteoporotic vertebral height loss remains challenging; deep learning (DL) models may improve detection and grading.

**Materials and methods:**

In this retrospective study, we evaluated eight human raters with different expertise (three students, three residents, and two attendings), four DL models, and one DL-based commercial software using the public Vertebral Segmentation (VerSe) 19 & 20 datasets. Vertebral fractures were graded using the semiquantitative Genant scale (0–3). Diagnostic performance was evaluated using interrater agreement and classification metrics, with significance tested via generalized linear mixed models across patient and vertebral levels for the thoracolumbar spine and its regional subsets. Consensus readings by a senior neuroradiologist and an experienced resident, informed by clinical data, served as the reference standard.

**Results:**

3548 thoracic and lumbar vertebrae from 331 patients were analyzed. 190 (5.4%) of vertebrae were fractured, and 139 (3.9%) had “clinically most relevant” moderate or severe fractures (Genant 2 or 3). DL models showed comparable accuracy as residents in detecting moderate/severe fractures (0.988 vs. 0.991, *p* > 0.05) on vetebral level. SpineQ v1.1 consistently showed comparable Area Under the Curve (AUROC) and higher diagnostic accuracy compared to experts in detecting any fracture and moderate/severe fractures across vertebral, regional, and patient-level analyses. Students consistently exhibited the lowest AUROC and diagnostic accuracy across all levels and comparisons.

**Conclusion:**

When specifically trained to detect a distinct condition like vertebral fractures, advanced algorithms can show comparable performance as experts.

**Key Points:**

***Question*** *Can dedicated deep learning-based algorithms improve diagnostic performance for accurate detection and grading of osteoporotic vertebral fractures on CT scans?*

***Findings*** *Deep learning models specifically trained for vertebral fracture detection and grading can reach comparable performance as experts for the identification and grading of osteoporotic fractures.*

***Clinical relevance*** *Specifically trained deep learning models represent a valuable advancement for improving the identification and grading of osteoporotic fractures in clinical practice, bringing these tools a significant step closer to routine clinical implementation.*

**Graphical Abstract:**

**Figure:**
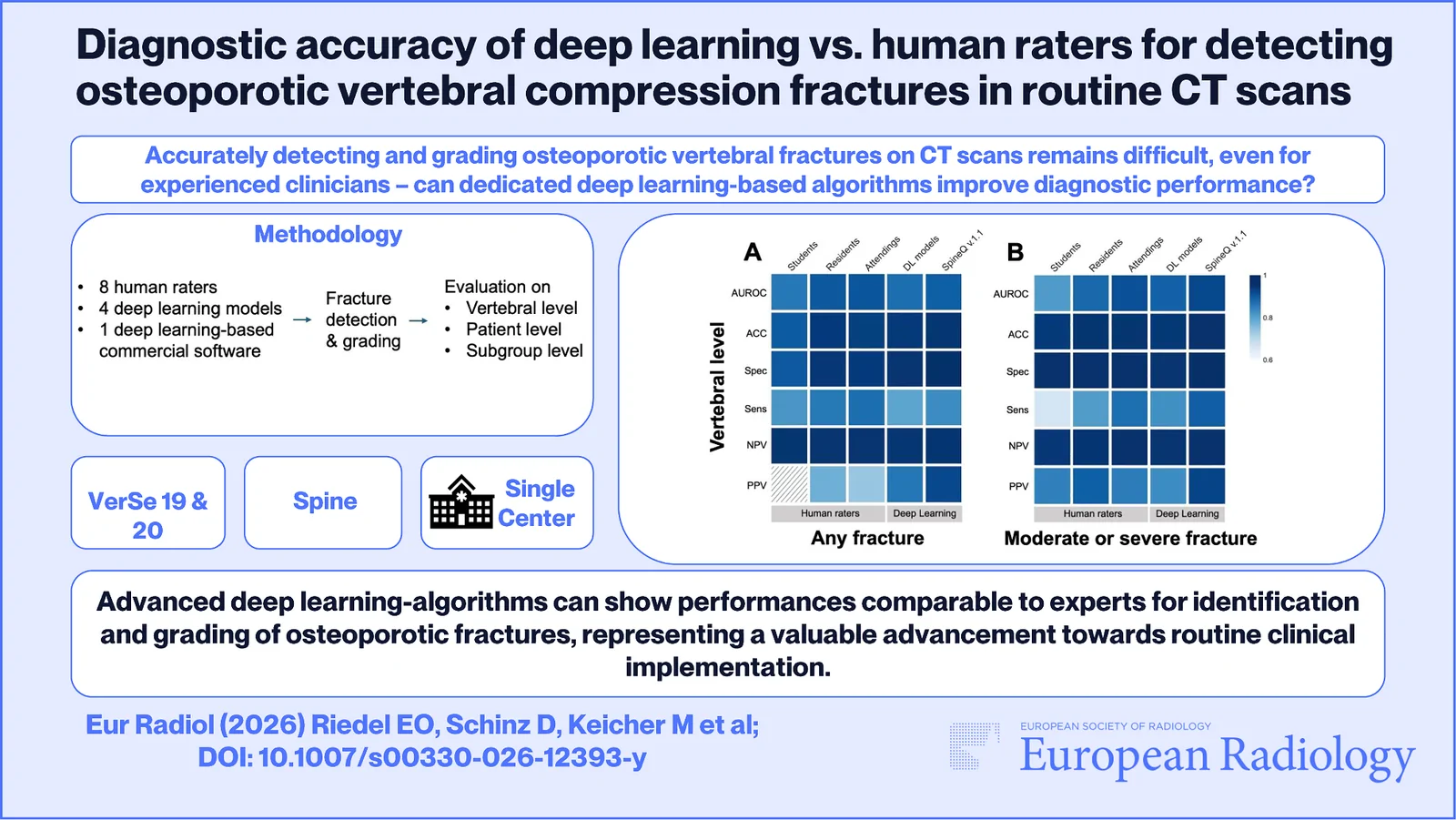

## Introduction

Osteoporosis is a systemic skeletal disorder characterized by decreased bone mass and deterioration of bone tissue, leading to increased bone fragility and susceptibility to fractures. Fragility fractures are a major public health issue, particularly among the elderly, due to their substantial impact on healthcare costs, loss of quality-adjusted life years, and significantly increased morbidity and mortality [1–4]. They are also associated with a markedly higher risk of subsequent fractures in both the short term [5–7] and long term [7–9]. Early identification of these fractures is crucial, as it can directly influence patient management by prompting the initiation of appropriate treatment [10]. Therefore, accurate diagnosis and inclusion of fragility fractures in radiologic reports are essential for improving patient outcomes. Next to dual x-ray absorptiometry, computed tomography imaging is a valuable tool for assessing bone quality and detecting osteoporotic fractures [11]. However, diagnosing osteoporotic fractures, especially mild fractures, using CT imaging can be challenging [12–14]. It is further complicated by differential diagnoses such as degenerative alterations or developmental anomalies, often leading to variability in assessments, especially among non-experts.

Deep learning algorithms have shown promise in improving the accuracy and efficiency of diagnosing osteoporotic fractures from CT images. As a subset of artificial intelligence (AI), deep learning (DL) employs multilayered neural networks to learn complex patterns from large datasets and has demonstrated remarkable success in image analysis tasks [15, 16]. In medical imaging, these algorithms can enhance diagnostic accuracy and efficiency by automating image interpretation and detecting subtle abnormalities that may be overlooked by human readers [17]. By analyzing large volumes of imaging data, they can identify subtle patterns and provide quantitative assessments that may be beyond the capability of human radiologists.

To assess the performance of deep learning algorithms relative to human raters and the conditions influencing it, we analyzed two outcomes at three levels using the semiquantitative Genant scale [18]: (1) the presence of any fracture (Genant 1–3) versus no fracture, and (2) the presence of a “clinically most relevant” moderate or severe fracture (Genant 2 or 3) versus a mild fracture (Genant 1) or no fracture on (i) patient level, (ii) single vertebral level, and (iii) single vertebral level by spinal region (upper, lower thoracic, and lumbar).

## Materials and methods

### Study design and ethics

In this retrospective diagnostic accuracy study, we evaluated four Deep Learning models (DL models), one DL-based commercial algorithm, and eight human raters (students, residents, attendings) using data from 3548 vertebrae across 331 patients from the Vertebral Segmentation (VerSe)19 and VerSe20 datasets [19–21]. Vertebral-level analysis was performed across the full spine and anatomical subsets (upper thoracic, lower thoracic, lumbar) to account for regionally different fracture prevalence, while patient-level analysis provided insight into clinically relevant individual fracture prevalence.

The institutional review board waived informed consent (Approval No. [27/19 S-SR]). No separate study protocol was registered. The study complies with the ethical standards of the 1964 Declaration of Helsinki and its later amendments [22].

### Training sets for deep learning models

For in-house training of the deep learning models, all patients from an internal CT dataset (813 patients, 1262 sessions, and 1635 scans, 24,219 vertebrae with 10.6% of vertebrae having any fracture and 9.1% of vertebrae having a Genant 2 or 3 fracture) were used for both training and validation through a 5-fold cross-validation approach, with one-fifth of the data used for validation in each fold. Vertebral centroid annotations with corresponding fracture ratings were provided, with approximately 20% of annotations contributed by each of J.S.K., N.S., T.B., S.R., and D.S. The dataset was acquired across a heterogeneous, multi-scanner environment, including different systems from Philips and Siemens. Images were reconstructed either axially with a slice thickness of less than 1.5 mm or sagittally, where the slice thickness ranged from 2 mm to 3 mm. Tube voltage settings were predominantly 120 kilovolt peak (kVp), with smaller proportions at 80 kVp, 100 kVp or 140 kVp. The dataset was collected from routine clinical CT examinations at our institution for patients aged > 45 years who underwent chest, abdomen, or pelvis scans between September 2008 and May 2017 in the outpatient clinic or during admission and had at least 5 years of prior medical records. Inclusion required sufficient thoracic and lumbar spine covering at least T6 to L4; patients with high-energy trauma fractures or multiple myeloma were excluded.

For SpineQ’s fracture detection development, a proprietary, multicenter CT fracture dataset comprising approximately 4000 CT scans was used. The cohort had a mean age of 64.8 years (standard deviation 13.6). The sex distribution was approximately 60% male and 40% female. The average number of fractures per scan was 0.25 in female scans and 0.19 in male scans. The dataset was acquired across a heterogeneous, multi-scanner environment, including systems from four major CT scanner manufacturers. Slice thickness ranged from 0.5 mm to 5 mm, with a mean slice thickness of 2.06 mm. Tube voltage settings were predominantly 120 kVp (95.5%), with smaller proportions from other voltages.

Neither of the two training sets contained any data from the VerSe datasets, used for evaluation in this study.

### Independent test set

For evaluation, we used data from the VerSe 19 & 20 datasets [19–21]. For these datasets, CT scans were obtained during clinical routine for a range of clinical indications, both related and unrelated to spine examination, such as acute or chronic back pain or suspected spinal fracture; cancer staging, restaging or follow-up; evaluation to exclude acute abdominal pathology; and postoperative assessment. CT scans were acquired at 120 kVp with thin-slice reconstructions (0.9–1 mm) or direction-specific resolutions up to 1.5 mm craniocaudal, 1 mm anterior–posterior, and 3 mm left-right. Images were reconstructed with a bone kernel (high-frequency reconstruction) to enhance cortical and trabecular bone detail, prioritizing sharpness over noise. Osseous alterations, including Schmorl nodes, hemangiomas, degenerative changes, and foreign materials from kyphoplasty or spondylodesis, were deliberately retained in the dataset to capture the broadest possible spectrum of spinal morphology [19–21]. Exclusion criteria were cervical vertebrae, as we focused on thoracic and lumbar vertebrae only, as well as anatomical variations (e.g., transitional vertebrae) and vertebrae for which complete ratings from all human raters were unavailable.

### Reference standard reading

To establish the reference standard, sagittal CT reformats were independently reviewed in ITK-SNAP [23] by an attending neuroradiologist (N.S.) with 9 years of experience and a neuroradiology resident (M.T.L.) with 4 years of extensive prior experience in vertebral fracture detection and rating. Both readers subsequently compared their assessments with clinical information available, and discrepancies were resolved through consensus reiteration, providing a reliable reference standard, and were not part of the final rater panel (*n* = 8). Fractures were graded using the semiquantitative Genant Scale [18]: 0 = no fracture, 1 = mild (20–25% height reduction), 2 = moderate (25–40% height reduction), and 3 = severe (> 40% height reduction). Degenerative Schmorl’s nodes and other non-fracture deformities, such as degenerative spondylosis, were rated only in the internal CT fracture dataset and grouped under category 0 for evaluation.

### Human ratings

Eight human raters—two neuroradiology attendings, three residents, and three final-year medical students—analyzed the scans independently while blinded to the patient data in ITK-SNAP with vertebral labels (e.g., Lumbar vertebra L1, L2) overlaid for consistency. The two attending radiologists (J.S.K., T.B.) had 20 and 14 years of experience, respectively; the three radiology residents (D.S., M.D., S.R.) had 1, 2, and 3 years of experience; and the three final-year medical students (L.M., I.S., A.W.) had completed approximately 3 months of specialized radiology training.

### Deep learning models

We trained four DL models on the internal CT fracture dataset: two specialized for vertebral fracture detection (Engstler et al [24] and Video-CT MAE [25]) and two general-purpose 3D Convolutional Neural Networks (CNNs) from the Medical Open Network for AI library (DenseNet121 [26] and SeResNet50 [27]). All models were trained for 100 epochs using cross-entropy loss and the AdamW optimizer. Models were initialized randomly and trained with a learning rate of 0.0005, except Video-CT MAE, which was fine-tuned with a learning rate of 0.00001 due to architectural specifics. Input volumes were resampled to 1 mm resolution, cropped to 96 × 96 × 96 voxels, and Hounsfield Units were cropped to the range [−1000, 1000] and scaled to [−1, 1]. Data augmentation included geometric and intensity transformations. Models classified vertebrae into six categories (no fracture, Genant 1–3, Schmorl’s node, and other deformities), with uniform class sampling to address imbalance. To ensure robust and generalizable model performance, we employed a 5-fold cross-validation strategy. For each architecture, five separate models were trained, one for each cross-validation fold. Within each fold’s training, the model that achieved the highest Mean Average Precision on the validation set was selected. These five models were then combined into a single ensemble model per architecture by averaging their predictions, resulting in four ensembles in total—one for each architecture. For subsequent statistical analysis, each ensemble was treated analogous to a single human rater, allowing the four DL ensembles to be grouped and compared to human rater groups.

Additionally, we evaluated one CE-marked (Conformité Européenne) commercial algorithm, SpineQ v.1.1 from Bonescreen GmbH, which is based on a deep learning algorithm with dedicated pre- and postprocessing, including image calibration.

### Statistical analysis

Analyses were conducted grouped as follows: students (*n* = 3), residents (*n* = 3), attendings (*n* = 2), DL models (*n* = 4) and SpineQ (*n* = 1). Cohen’s kappa and weighted kappa, Accuracy, Sensitivity (Sens), Specificity (Spec), Positive Predictive Value (PPV), and Negative Predictive Value (NPV) as well as Area under the receiver operating characteristic curve (AUROC) were calculated per group in R v4.2.2 with the packages irr v0.84.1, epiR v2.0.80, caret v7.0.1 and pROC v1.18.5. For comparability, AUROC was computed using raw ordinal ratings (0–3) as continuous severity scores from both human raters and DL models. Ninety-five percent confidence intervals (95% CIs) for proportions were derived using the Wilson method. Cohen’s kappa was interpreted according to McHugh et al [28]. To compare group performance, we fitted binomial Generalized Linear Mixed Models (GLMMs) using R v4.2.2 with the packages lme4 v1.1.37 and emmeans v1.11.1. Vertebra-level performance was analyzed with binomial GLMMs including Group as a fixed effect and random intercepts for vertebra and patient (Correct ~ Group + (1 | Vertebra_ID) + (1 | Patient_ID)). We also tested adjustment for anatomical level (Vertebra_Group) and inclusion of rater as an additional random intercept at the vertebral level and at the vertebral level in the lumbar subgroup; neither improved model fit (level: Likelihood-Ratio Test *p* = 0.75; rater variance ≈ 0), so they were not included in the final model. Patient-level analyses used binomial GLMMs with random intercept for patient: Correct ~ Group + (1 | Patient_ID). We tested inclusion of a rater random intercept (1 | Rater_ID); rater variance was negligible and did not improve fit, so the parsimonious model was used. Post hoc comparisons were Bonferroni-corrected. An adjusted *p*-value < 0.05 was considered significant. Visualizations were created using R v4.2.2 with the package ggplot2 v3.5.2 and BioRender for heatmap visualizations.

## Results

### Study cohort

**Fig. 1:**
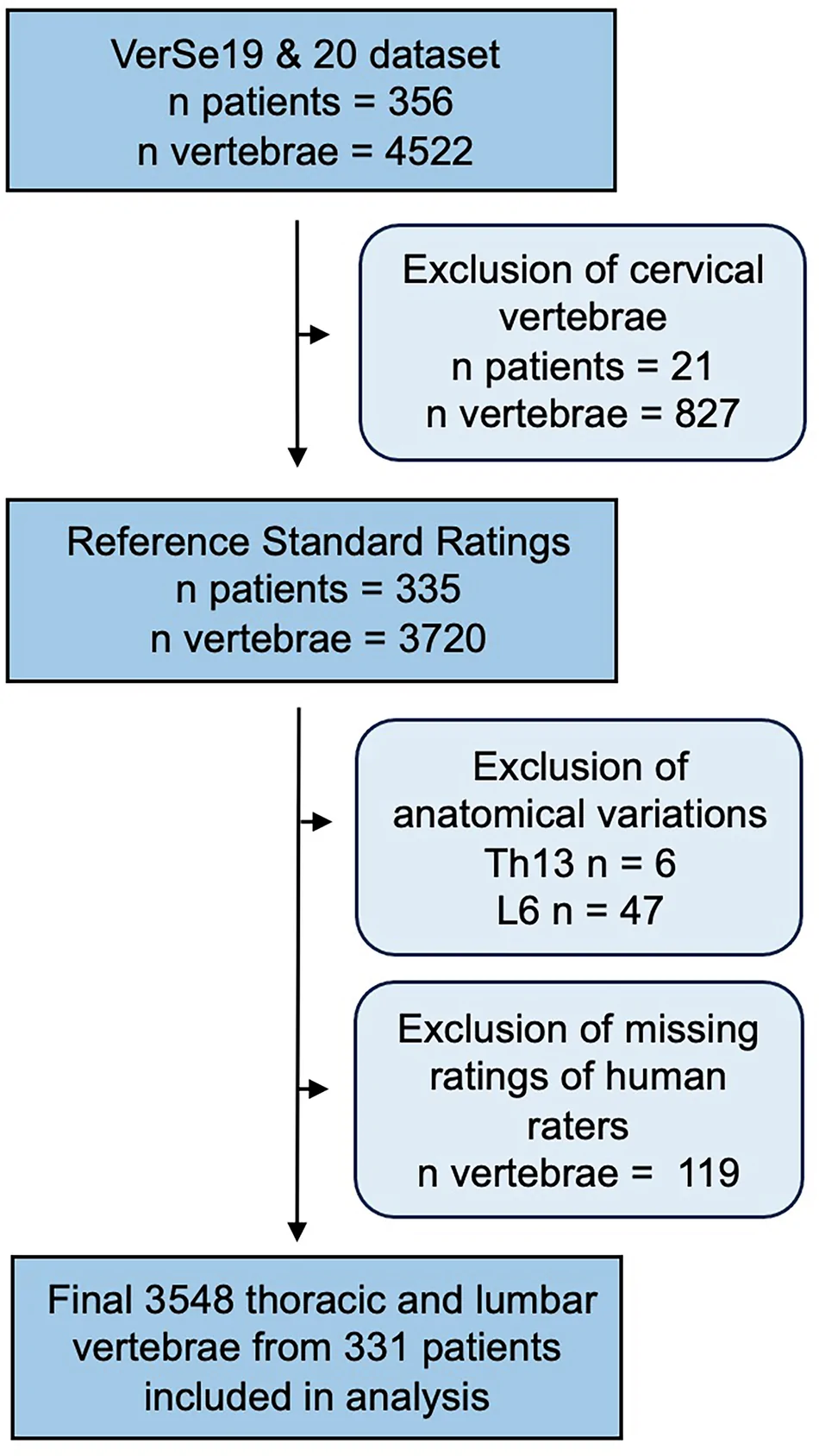
Exclusion flowchart. VerSe, Vertebral segmentation

From a total of 4522 vertebrae across 356 patients in the VerSe 19 and 20 datasets, 827 cervical vertebrae from 21 patients were excluded as the analysis focused on thoracic and lumbar regions, yielding reference standard readings for 3720 vertebrae in 335 patients. Subsequently, 53 vertebrae were excluded due to anatomical variations (e.g., transitional vertebrae), and 119 vertebrae were excluded because complete ratings from all raters were unavailable (Fig. 1). The final independent test cohort included 331 patients: 45% male, 50% female, and 5% with missing sex data. The mean age was 59.5 ± 17.5 years (min. =18.9, max. = 90.6, 75.8% ≥ 50 years). 57.49 ± 16.81 years (min = 18.9, max = 89.3, 70.0% ≥ 50 years) for males and 61.33 ± 17.95 years (min =19.0, max = 90.6, 78.7% ≥ 50 years) for females. CT scans were acquired using various scanners from Philips (ICT 12.4% and IQON 28.9%), Siemens (Somatom AS + 17.1%, Somatom AS 1.3%, Biograph 64 2.9%, Sensation Cardiac 64 1.0% and other 8.3%), GE systems (6.0%), and Toshiba systems (6.0%). 49.5% of scans were performed without contrast, 8.6% during arterial, 41.0% during portal venous, and 1.0% during late portal venous phase.

### Distribution of vertebral fractures

**Fig. 2:**
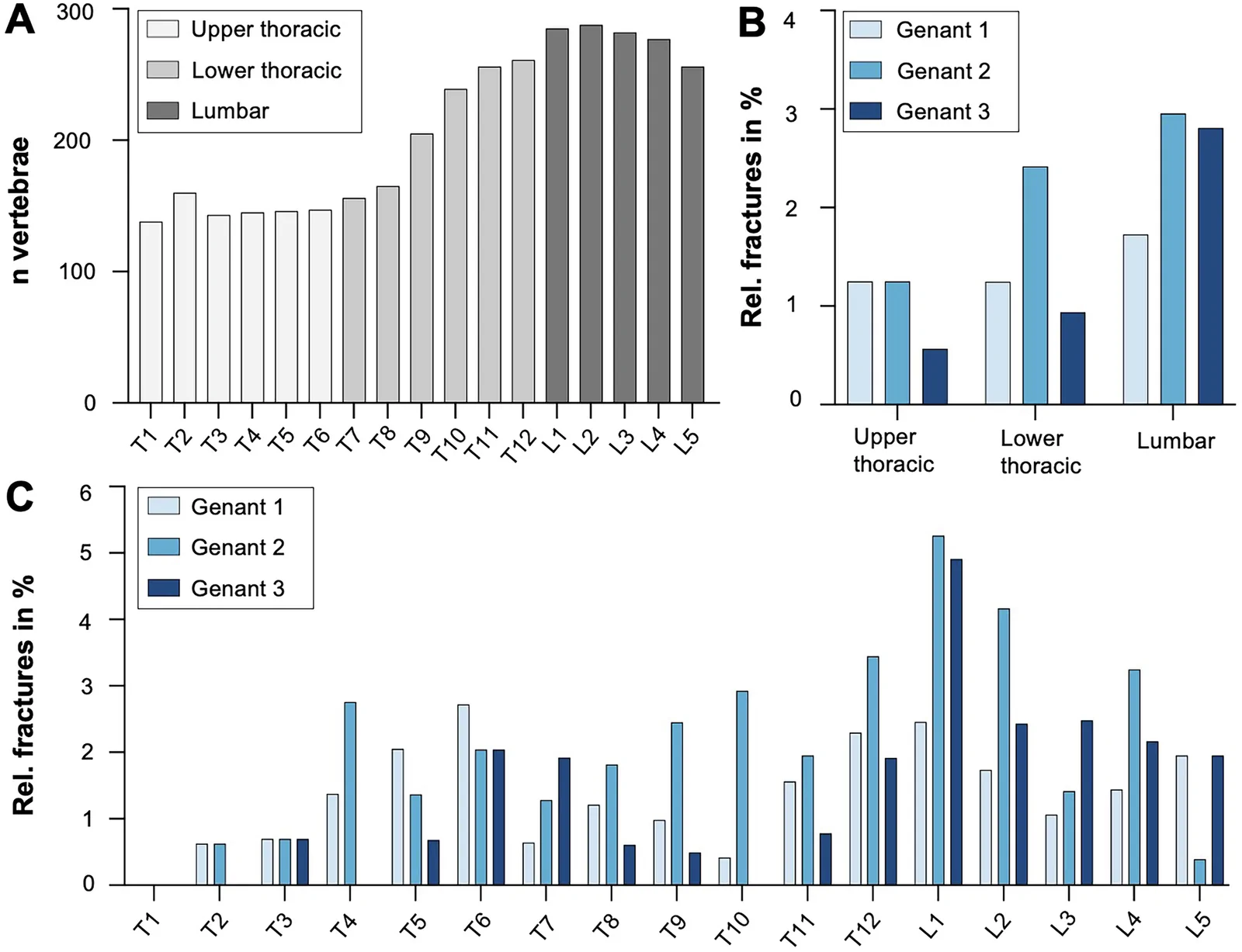
Vertebrae and fracture distribution. **A** Distribution of vertebrae. **B** Relative fractures per subset: upper thoracic vertebrae (T1–T6), lower thoracic vertebrae (T7–T12), and lumbar vertebrae (L1–L5). **C** Relative fractures per single vertebra

We analyzed 3548 thoracic and lumbar vertebrae from 331 patients, grouped into upper thoracic (thoracic vertebrae T1–T6), lower thoracic (T7–T12), and lumbar (L1–L6) subregions (Fig. 2A). At the patient level, 85 (25.7%) had at least one Genant 1 fracture and 74 (22.4%) had at least one moderate or severe fracture. Of the 3548 vertebrae, 190 (5.4%) showed any osteoporotic fracture, and 139 (3.9%) had a “clinically most relevant” (Genant 2 or 3) fracture. Relative fracture prevalence increased from the upper thoracic vertebrae over the lower thoracic vertebrae to the lumbar vertebrae. Of the 879 upper thoracic vertebrae, 3.1% (27) had a fracture of any grade, and 1.8% (16) had a moderate or severe fracture, with each 1.3% (11) Genant grade 1 and 2 fractures and 0.6% (5) Genant grade 3 fractures. Of the 1281 lower thoracic vertebrae, 4.6% (59) had any fracture and 3.4% (43) had a moderate or severe fracture, with 1.3% (16) grade 1, 2.4% (31) grade 2 and 0.9% (12) grade 3 fractures. Out of 1388 lumbar vertebrae, 7.5% (104) had any fracture, and 5.8% (80) had moderate or severe fractures, with 1.7% (24) grade 1, 2.9% (41) grade 2 and 2.8% (39) grade 3 fractures (Fig. 2B). Fracture distribution per single vertebra is shown in Fig. 2C.

### Diagnostic performance at the vertebral level

**Fig. 3:**
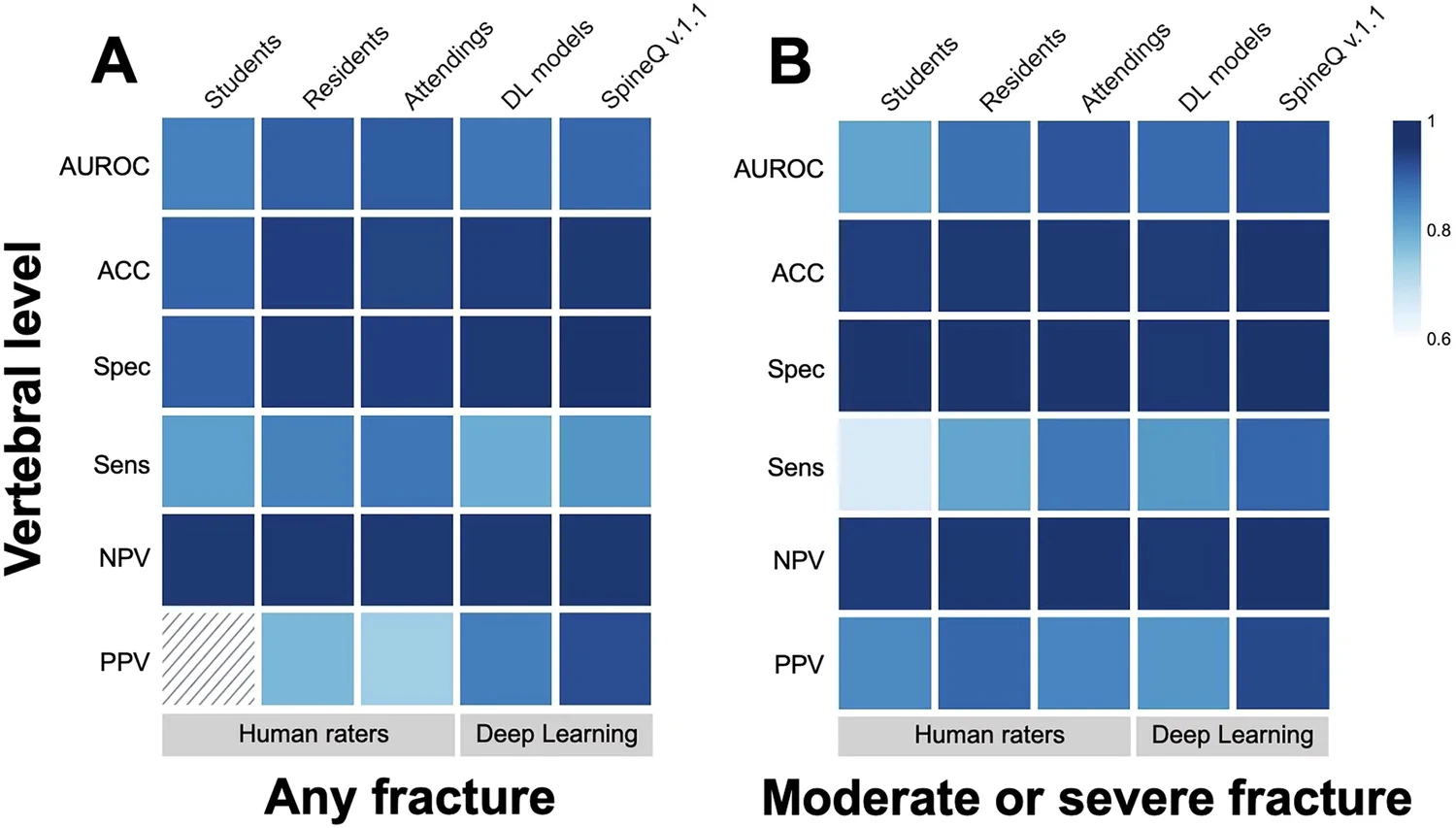
Diagnostic performance on the vertebral level. Heatmaps representing diagnostic performance for (**A**) discrimination of any fracture (Genant 1–3) from no fracture (Genant 0) and (**B**) differentiation of a “clinically most relevant” moderate or severe fracture (Genant 2 or 3) from no or mild fracture (Genant 0 or 1) on vertebral level. ACC, accuracy; NPV, negative predictive value; PPV, positive predictive value; Sens, sensitivity; Spec, Specificity

Diagnostic performance metrics at the vertebral level for the whole spine for detecting any fracture (Genant 1–3 vs. Genant 0) is shown in Table 1A and Fig. 3A. AUROC was comparable for residents, attendings and SpineQ (0.928–0.944), and lower for DL models (0.906) and even lower for students (0.890). Accuracy was highest for SpineQ (0.990), followed by DL models (0.985), residents (0.982) and attendings (0.977). SpineQ and DL models were more specific, and human raters more sensitive. NPV was comparably high for all groups (< 0.99). PPV was highest for SpineQ (0.962) and DL models (0.894) and lower for all human raters. GLMM was used to assess statistical differences between groups, where SpineQ v1.1 achieved significantly better results than DL models (*p*_adj_ = 0.0022), and attendings, residents, and students (*p*_adj_ < 0.0001). Attendings performed significantly better than DL models (*p*_adj_ < 0.0001), while students performed considerably worse than all groups (*p*_adj_ < 0.0001).

**Table 1:** Diagnostic performance (means of point estimates with 95% confidence intervals calculated using the Wilson method) for fracture detection of **A** fracture versus no fracture (Genant 1, 2 or 3 fracture versus no fracture), as well as **B** moderate or severe fracture (Genant 2 or 3) versus mild or no fracture (Genant 1 or 0) on vertebral level

Metric	Students	Residents	Attendings	DL models	SpineQ v.1.1
Weighted κ	0.634 (0.609–0.659)	0.829 (0.811–0.847)	0.839 (0.817–0.862)	0.825 (0.808–0.842)	0.907 (0.890–0.924)
**(A) Vertebral level 0 vs. 123**					
Cohen’s κ	0.573 (0.545–0.601)	0.831 (0.808–0.855)	0.802 (0.773–0.832)	0.846 (0.825–0.867)	0.903 (0.879–0.926)
AUROC	0.890 (0.875–0.904)	0.938 (0.925–0.951)	0.944 (0.929–0.958)	0.906 (0.893–0.920)	0.928 (0.911–0.945)
Accuracy	0.932 (0.927–0.937)	0.982 (0.979–0.984)	0.977 (0.974–0.980)	0.985 (0.983–0.987)	0.990 (0.988–0.993)
Spec	0.937 (0.932–0.941)	0.987 (0.985–0.989)	0.982 (0.978–0.985)	0.994 (0.993–0.996)	0.998 (0.997–0.999)
Sens	0.843 (0.813–0.873)	0.888 (0.861–0.915)	0.906 (0.857–0.936)	0.818 (0.790–0.846)	0.859 (0.822–0.896)
NPV	0.991 (0.989–0.992)	0.994 (0.992–0.995)	0.994 (0.993–0.996)	0.990 (0.988–0.992)	0.992 (0.990–0.994)
PPV	0.502 (0.471–0.534)	0.798 (0.766–0.830)	0.754 (0.715–0.792)	0.894 (0.870–0.918)	0.962 (0.939–0.985)
**(B) Vertebral level 01 vs. 23**					
Cohen’s κ	0.748 (0.713–0.783)	0.872 (0.847–0.897)	0.889 (0.861–0.916)	0.847 (0.824–0.870)	0.947 (0.928–0.966)
AUROC	0.833 (0.811–0.856)	0.914 (0.896–0.932)	0.950 (0.932–0.967)	0.923 (0.908–0.937)	0.964 (0.949–0.979)
Accuracy	0.983 (0.980–0.986)	0.991 (0.989–0.993)	0.991 (0.989–0.994)	0.988 (0.987–0.990)	0.996 (0.995–0.998)
Spec	0.996 (0.994–0.997)	0.997 (0.996–0.998)	0.995 (0.993–0.997)	0.994 (0.993–0.995)	0.999 (0.998–1.000)
Sens	0.671 (0.625–0.718)	0.831 (0.793–0.869)	0.904 (0.867–0.941)	0.852 (0.821–0.883)	0.929 (0.896–0.961)
NPV	0.987 (0.984–0.989)	0.993 (0.991–0.995)	0.996 (0.994–0.998)	0.994 (0.993–0.995)	0.997 (0.996–0.999)
PPV	0.876 (0.838–0.913)	0.928 (0.900–0.957)	0.883 (0.844–0.922)	0.856 (0.825–0.887)	0.970 (0.947–0.994)

For comparability, AUROC was computed using raw ordinal ratings (0–3) as continuous severity scores from both human raters and DL models

For detecting moderate/severe fractures (Genant 2–3 vs. Genant 0–1), results are shown in Table 1B and Fig. 3B. AUROC was highest for SpineQ (0.964) and attendings (0.950), followed by DL models and residents (0.923 and 0.914). Accuracy was also highest for SpineQ and attendings and residents (> 0.99), followed by DL models and students (> 0.98). While specificity was comparably high for all groups (> 0.99), sensitivity was highest for SpineQ (0.929) and attendings (0.904). NPV was > 0.99 for all groups except for students and PPV was highest for SpineQ (0.970). GLMM was applied to compare group ratings. Again, SpineQ v1.1 demonstrated superior performance to all other groups: DL models (*p*_adj_ < 0.0001), attendings (*p*_adj_ = 0.0001), residents and students (*p*_adj_ < 0.0001).

Weighted κ and Cohen’s κ for both levels of comparison showed almost perfect agreement with the reference standard for SpineQ (all κ > 0.9) and DL models, residents and attendings (all κ > 0.8).

Overall, at the vertebral level across the entire spine, SpineQ v1.1 consistently achieved the highest diagnostic performance across tasks, outperforming DL models (*p*_adj_ ≤ 0.0022) and all reader groups (*p*_adj_ ≤ 0.0001). For detecting any fracture, AUROC was comparable for SpineQ, attendings, and residents (≈ 0.93–0.94), but lower for DL models and students; accuracy and PPV were highest for SpineQ and DL models, while human raters were more sensitive. For moderate/severe fractures, SpineQ and attendings showed the best AUROC (>0.95) and accuracy (> 0.99), with SpineQ significantly superior to all other groups (*p*_adj_ ≤ 0.0001).

**Fig. 4:**
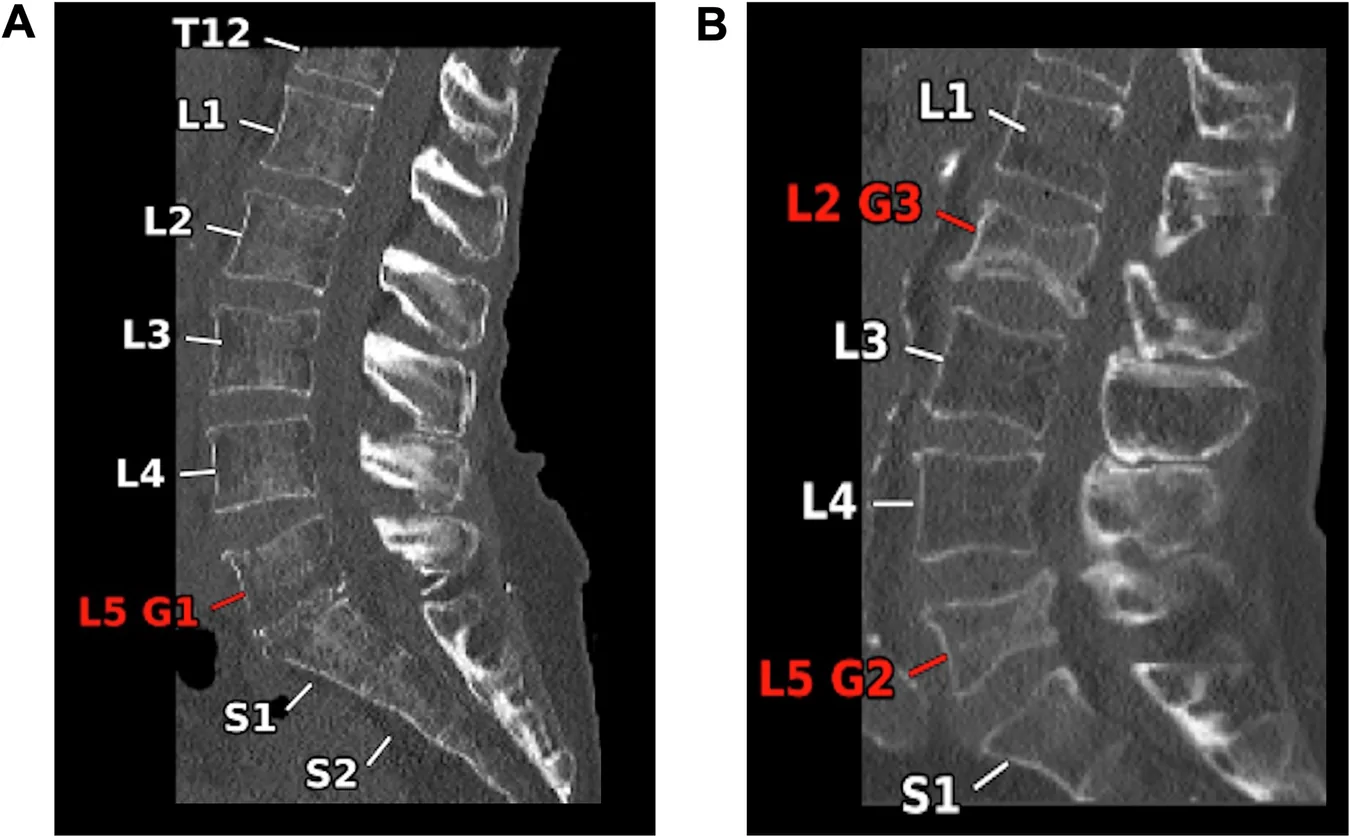
Examples of incorrectly identified fractures. **A** A grade 1 fracture, which was overlooked by two of the three students and three of the four deep learning (DL) models. One DL model and one attending physician incorrectly classified it as a Genant grade 2 fracture. **B** Genant grade 3 fractures in L2 and L5 were misclassified as grade 2 by one attending, all residents, and two students. Additionally, the L5 fracture was not detected by any of the DL models. G = Genant grade

To assess model performance in particularly challenging cases, we analyzed vertebrae for which the reference standard required consensus review. Among 3548 vertebrae, 116 (3.3%) involved disagreement, while 3433 (96.7%) showed reader agreement. In the disagreement group, 88 vertebrae had no fractures, 11 had grade 1, 10 had grade 2, and 7 had grade 3 fractures. For these difficult cases, mean DL models achieved 84.1% accuracy (95% CI: 80.4–87.3%) compared to 97.5% (95% CI: 97.3–97.8%) in agreement cases, with overall accuracy of 97.1% (95% CI: 96.8–97.4%; *p* = 1.03 × 10⁻⁶³). SpineQ showed 91.4% accuracy (95% CI: 84.7–95.8%) in disagreement cases and 97.5% (95% CI: 97.3–97.8%) in agreement cases, with overall accuracy of 97.1% (95% CI: 96.8–97.4%; *p* = 3.38 × 10⁻⁸). Although performance was lower in consensus cases, accuracy remained high even for these challenging vertebrae. Figure 4A shows an example of a Genant grade 1 fracture, and Fig. 4B shows an example of a Genant grade 3 fracture that was partially not correctly identified by the raters.

### Diagnostic performance at the vertebral level for upper, lower thoracic, and lumbar subsets

#### Upper thoracic vertebrae

**Fig. 5:**
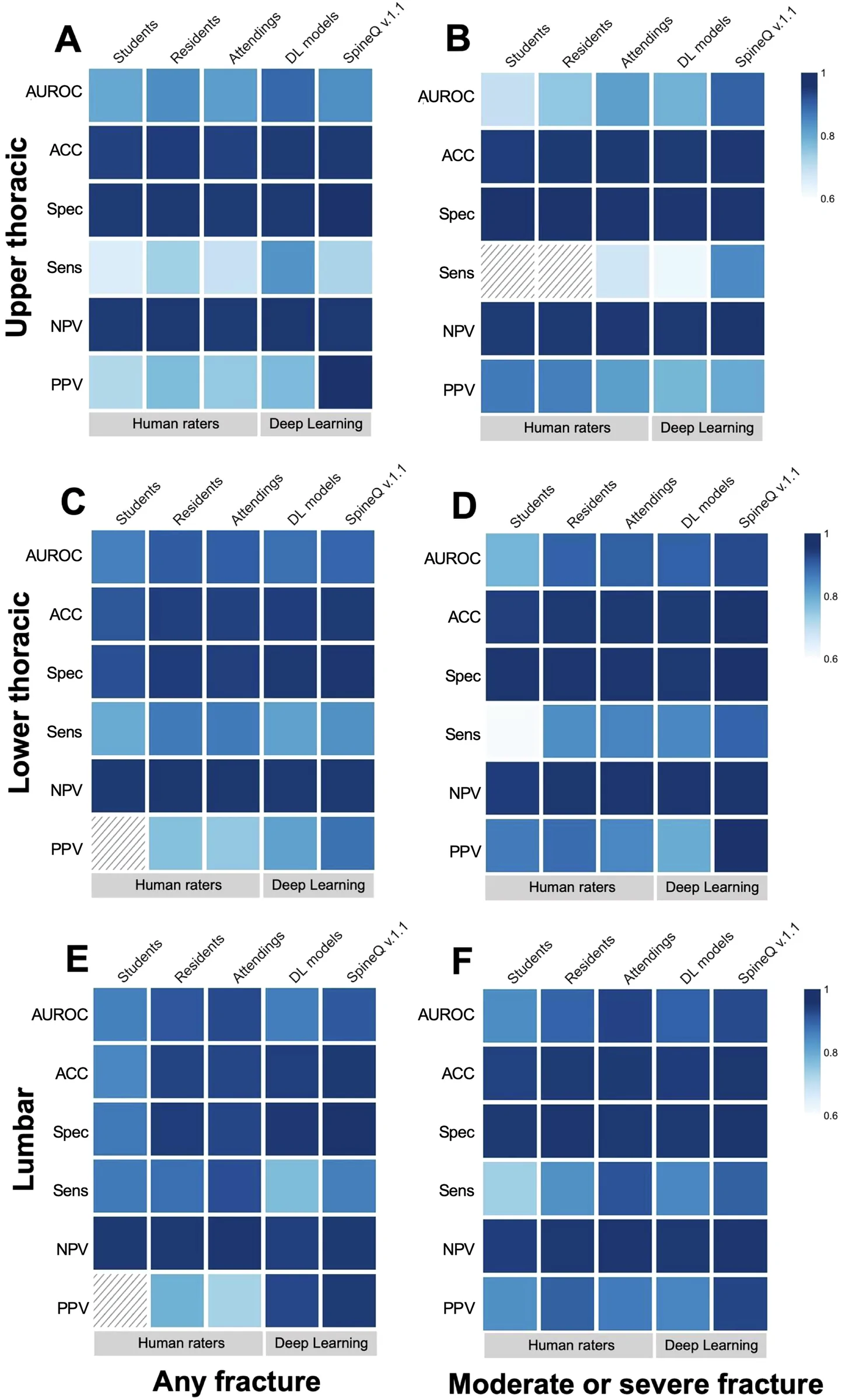
Diagnostic performance in vertebral level subsets. Heatmaps representing diagnostic performance for discrimination of any fracture (Genant 1–3) from no fracture (Genant 0) and differentiation of a “clinically most relevant” moderate or severe fracture (Genant 2 or 3) from no or mild fracture (Genant 0 or 1) on vertebral level in the subsets of (**A**, **B**) upper thoracic vertebrae, (**C**, **D**) lower thoracic vertebrae, and (**E**, **F**) lumbar vertebrae. ACC, accuracy; NPV, negative predictive value; PPV, positive predictive value; Sens, sensitivity; Spec, Specificity

Diagnostic performance metrics for identifying any fracture (Genant grades 1–3) versus no fracture in the upper thoracic subset are presented in Table 2A and Fig. 5A. AUROC was highest for DL models (0.927) and SpineQ and residents (> 0.87). Accuracies were highest for SpineQ (0.992), followed by all other groups (> 0.98). SpineQ showed the highest specificity with 1.00 and DL models the highest sensitivity with 0.86. NPV was high for all groups (> 0.98) while PPV was considerably highest for SpineQ with 1.00 (all other groups <0.80). Comparing group performances using GLMM, SpineQ v1.1 demonstrated significantly superior performance compared to attendings (*p*_adj_ = 0.0080) and students (*p*_adj_ = 0.0014). Additionally, DL models outperformed students (*p*_adj_ = 0.0100).

**Table 2:** Diagnostic performance (means of point estimates with 95% confidence intervals calculated using the Wilson method) on single vertebral level in the subset of upper thoracic vertebrae (**A**, **B**) as well as in the subset of lower thoracic vertebrae (**C**, **D**) for discrimination of any fracture (Genant 1–3) from no fracture (Genant 0) and differentiation of a “clinically most relevant” moderate or severe fracture (Genant 2 or 3) from no or mild fracture (Genant 0 or 1)

Metric	Students	Residents	Attendings	DL models	SpineQ v.1.1
**Weighted Cohen’s κ**					
Upper thoracic	0.657 (0.577–0.737)	0.750 (0.680–0.819)	0.733 (0.640–0.825)	0.780 (0.781–0.909)	0.845 (0.781–0.909)
Lower thoracic	0.649 (0.604–0.694)	0.850 (0.818–0.881)	0.842 (0.801–0.884)	0.836 (0.806–0.865)	0.922 (0.892–0.952)
**(A) Vertebral level upper thoracic 0 vs. 123**					
Cohen’s κ	0.664 (0.577–0.750)	0.760 (0.686–0.834)	0.682 (0.578–0.787)	0.819 (0.764–0.874)	0.847 (0.768–0.926)
AUROC	0.829 (0.767–0.890)	0.873 (0.818–0.929)	0.847 (0.777–0.917)	0.927 (0.888–0.965)	0.870 (0.800–0.940)
Accuracy	0.980 (0.975–0.986)	0.986 (0.981–0.991)	0.981 (0.974–0.988)	0.989 (0.985–0.993)	0.992 (0.987–0.997)
Spec	0.990 (0.986–0.994)	0.993 (0.990–0.996)	0.990 (0.985–0.995)	0.992 (0.989–0.996)	1.000 (0.999–1.000)
Sens	0.667 (0.562–0.772)	0.753 (0.655–0.852)	0.704 (0.585–0.823)	0.861 (0.789–0.933)	0.741 (0.617–0.865)
NPV	0.989 (0.985–0.994)	0.992 (0.988–0.996)	0.990 (0.985–0.996)	0.995 (0.993–0.998)	0.992 (0.988–0.996)
PPV	0.732 (0.633–0.830)	0.795 (0.700–0.890)	0.766 (0.654–0.879)	0.794 (0.717–0.872)	1.000 (0.941–1.000)
**(B) Vertebral level upper thoracic 01 vs. 23**					
Cohen’s κ	0.559 (0.420–0.698)	0.665 (0.543–0.787)	0.747 (0.624–0.870)	0.697 (0.545–0.908)	0.846 (0.752–0.940)
AUROC	0.708 (0.614–0.802)	0.770 (0.682–0.858)	0.842 (0.745–0.940)	0.811 (0.738–0.884)	0.936 (0.869–1.000)
Accuracy	0.988 (0.984–0.993)	0.991 (0.987–0.995)	0.992 (0.987–0.996)	0.990 (0.987–0.994)	0.994 (0.990–0.998)
Spec	0.999 (0.997–1.000)	0.999 (0.997–1.000)	0.997 (0.994–1.000)	0.997 (0.995–0.999)	0.997 (0.994–1.000)
Sens	0.417 (0.273–0.561)	0.542 (0.399–0.685)	0.688 (0.519–0.857)	0.625 (0.502–0.748)	0.875 (0.745–1.000)
NPV	0.990 (0.985–0.994)	0.992 (0.988–0.996)	0.994 (0.990–0.998)	0.993 (0.990–0.996)	0.998 (0.995–1.000)
PPV	0.900 (0.758–1.000)	0.894 (0.759–1.000)	0.844 (0.705–0.983)	0.803 (0.864–0.921)	0.824 (0.684–0.964)
**(C) Vertebral level lower thoracic 0 vs. 123**					
Cohen’s κ	0.624 (0.572–0.676)	0.828 (0.787–0.869)	0.808 (0.755–0.861)	0.829 (0.792–0.866)	0.884 (0.840–0.928)
AUROC	0.891 (0.859–0.923)	0.944 (0.804–1.000)	0.942 (0.911–0.973)	0.915 (0.887–0.942)	0.931 (0.895–0.967)
Accuracy	0.954 (0.947–0.960)	0.983 (0.979–0.987)	0.980 (0.975–0.986)	0.985 (0.981–0.988)	0.990 (0.986–0.994)
Spec	0.960 (0.953–0.966)	0.987 (0.983–0.991)	0.984 (0.979–0.990)	0.992 (0.989–0.995)	0.996 (0.993–0.999)
Sens	0.822 (0.767–0.878)	0.900 (0.852–0.948)	0.900 (0.842–0.958)	0.838 (0.788–0.887)	0.867 (0.801–0.933)
NPV	0.991 (0.988–0.994)	0.995 (0.992–0.998)	0.995 (0.992–0.998)	0.992 (0.989–0.995)	0.993 (0.989–0.997)
PPV	0.589 (0.530–0.648)	0.784 (0.726–0.842)	0.768 (0.700–0.837)	0.838 (0.788–0.888)	0.912 (0.854–0.970)
**(D) Vertebral level lower thoracic 01 vs. 23**					
Cohen’s κ	0.709 (0.641–0.778)	0.892 (0.851–0.932)	0.879 (0.828–0.929)	0.842 (0.802–0.883)	0.963 (0.934–0.992)
AUROC	0.805 (0.754–0.856)	0.934 (0.901–0.968)	0.941 (0.902–0.980)	0.937 (0.909–0.965)	0.966 (0.935–0.997)
Accuracy	0.984 (0.979–0.988)	0.993 (0.990–0.996)	0.992 (0.988–0.996)	0.989 (0.986–0.992)	0.998 (0.996–1.000)
Spec	0.997 (0.995–0.999)	0.997 (0.995–0.999)	0.996 (0.992–0.999)	0.993 (0.991–0.996)	1.000 (0.999–1.000)
Sens	0.614 (0.529–0.698)	0.871 (0.809–0.934)	0.886 (0.812–0.960)	0.881 (0.829–0.932)	0.932 (0.871–0.993)
NPV	0.986 (0.982–0.990)	0.995 (0.993–0.998)	0.996 (0.993–0.999)	0.996 (0.993–0.999)	0.998 (0.996–1.000)
PPV	0.899 (0.834–0.965)	0.921 (0.867–0.975)	0.882 (0.811–0.953)	0.822 (0.811–0.953)	1.000 (0.970–1.000)

For distinguishing moderate or severe fractures (Genant 2 or 3) from mild or no fractures, results are shown in Table 2B and Fig. 5B. In this task, SpineQ had the highest AUROC with 0.936, followed by attendings (0.842) and DL models (0.811). Accuracy was highest for SpineQ (0.994) with values > 0.98 for all groups. All groups were highly specific (*p* > 0.99), with the highest sensitivity for SpineQ (0.875). NPV was high for all groups (> 0.99) and PPV was higher for all human raters than for DL models and SpineQ. Comparing group performances using GLMM, SpineQ v1.1 again showed a statistically significant advantage over students (*p*_adj_ = 0.0173).

Weighted Cohen’s κ showed almost perfect agreement with the reference standard for SpineQ (0.845). Cohen’s κ demonstrated almost perfect agreement for SpineQ for both levels of comparison (> 0.84) and for DL models for detection of any versus no fractures (0.819).

#### Lower thoracic vertebrae

Performance metrics for detecting any fracture in the lower thoracic region are detailed in Table 2C and Fig. 5C. AUROC was highest for residents and attendings (> 0.94), followed by SpineQ (0.93). Accuracy was highest for SpineQ (0.99), followed by DL models, residents and attendings (> 0.98). SpineQ and DL models were most specific with values > 0.99 and residents and attendings were most sensitive (0.90). NPV was high for all groups (> 0.99) and PPV highest for SpineQ (0.912) and DL models (0.84). GLMM, which was applied to compare group ratings, showed that SpineQ v1.1 significantly surpassed both attendings (*p*_adj_ = 0.0236) and students (*p*_adj_ < 0.0001), with students performing markedly worse than all other groups (*p*_adj_ < 0.0001).

For the classification of moderate/severe versus mild or no fractures (Table 2D, Fig. 5D), AUROC was highest for SpineQ (0.966), followed by attendings (0.941) and residents and ML models (> 0.93). Accuracy was high with values > 0.99 for SpineQ, residents and attendings and > 0.98 for DL models and students. Specificity and sensitivity were both highest for SpineQ (1.000 and 0.932 respectively). NPV was highest for Spine Q with 0.998 and values > 0.98 for all groups. PPV was with 1.00 highest for SpineQ. In group comparisons, using GLMM, SpineQ v1.1 achieved significantly better results than DL models (*p* = 0.0002), attendings (*p*_adj_ = 0.0175), and students (*p*_adj_ < 0.0001), and showed a trend toward outperforming residents (*p*_adj_ = 0.0566). Students again underperformed significantly compared to SpineQ v1.1 and residents (*p*_adj_ < 0.0001) and attendings (*p*_adj_ = 0.0032).

Weighted Cohen’s κ (SpineQ > 0.9 and DL models, residents and attendings all κ > 0.8) and Cohen’s κ for both levels of comparison showed almost perfect agreement to the reference standard (SpineQ > 0.9 for classification of moderate/severe versus mild or no fractures, all other κ for SpineQ, DL models, residents and attendings > 0.8).

#### Lumbar vertebrae

Table 3A and Fig. 5E provide metrics for detecting any fracture of the lumbar subset. AUROC was highest for attendings (0.967), followed by residents and SpineQ (≈ 0.95). Accuracy was highest for SpineQ (0.991). Specificity was highest for SpineQ and DL models (0.999 and 0.994 respectively). Sensitivity was higher for all human rater groups than for SpineQ and DL models. NPV was highest for attendings (0.998) with high performances for all groups (> 0.99, except DL models with 0.983). PPV was highest for SpineQ (0.989), followed by DL models (0.971). In group comparisons with GLMM, SpineQ v1.1 significantly achieved better results than all other groups: attendings (*p*_adj_ < 0.0001), residents (*p*_adj_ = 0.0001), and DL models (*p*_adj_ = 0.0156). DL models also showed a significant advantage over attendings (*p*_adj_ = 0.0047). Students consistently performed the worst among all groups (*p*_adj_ < 0.0001).

**Table 3:** Diagnostic performance (means of point estimates with 95% confidence intervals calculated using the Wilson method) for (**A**) discrimination of any fracture (Genant 1–3) from no fracture (Genant 0) and (**B**) differentiation of a “clinically most relevant” moderate or severe fracture (Genant 2 or 3) from no or mild fracture (Genant 0 or 1) on single vertebral level in the subset of lumbar vertebrae

Metric	Students	Residents	Attendings	DL models	SpineQ v.1.1
Weighted κ	0.617 (0.585–0.649)	0.832 (0.810–0.855)	0.856 (0.829–0.883)	0.828 (0.805–0.851)	0.899 (0.876–0.922)
**(A) Vertebral Level Lumbar 0 vs. 123**					
Cohen’s κ	0.525 (0.489–0.561)	0.849 (0.820–0.879)	0.824 (0.785–0.862)	0.863 (0.837–0.890)	0.935 (0.909–0.961)
AUROC	0.890 (0.874–0.984)	0.950 (0.932–0.968)	0.967 (0.951–0.983)	0.896 (0.872–0.919)	0.947 (0.923–0.971)
Accuracy	0.881 (0.871–0.891)	0.978 (0.973–0.983)	0.972 (0.962–0.978)	0.983 (0979–0.986)	0.991 (0.987–0.995)
Spec	0.903 (0.894–0.912)	0.988 (0.985–0.992)	0.972 (0.965–0.979)	0.994 (0.992–0.997)	0.999 (0.998–1.000)
Sens	0.900 (0.865–0.935)	0.917 (0.884–0.950)	0.962 (0.932–0.991)	0.793 (0.752–0.834)	0.894 (0.849–0.939)
NPV	0.991 (0.988–0.995)	0.993 (0.990–0.996)	0.998 (0.996–1.000)	0.983 (0.980–0.987)	0.991 (0.987–0.995)
PPV	0.447 (0.407–0.486)	0.813 (0.770–0.855)	0.749 (0.696–0.802)	0.971 (0.949–0.992)	0.989 (0.968–1.000)
**(B) Vertebral level Lumbar 01 vs. 23**					
Cohen’s κ	0.793 (0.751–0.835)	0.894 (0.864–0.925)	0.921 (0.889–0.952)	0.875 (0.848–0.903)	0.953 (0.928–0.978)
AUROC	0.873 (0.841–0.906)	0.932 (0.906–0.957)	0.975 (0.957–0.993)	0.937 (0.916–0.958)	0.968 (0.946–0.990)
Accuracy	0.979 (0.970–0.986)	0.989 (0.986–0.992)	0.991 (0.987–0.995)	0.986 (0.983–0.990)	0.995 (0.992–0.998)
Spec	0.993 (0.990–0.996)	0.996 (0.994–0.999)	0.993 (0.989–0.997)	0.993 (0.990–0.995)	0.998 (0.996–1.000)
Sens	0.754 (0.697–0.811)	0.867 (0.821–0.913)	0.956 (0.920–0.993)	0.882 (0.843–0.920)	0.938 (0.896–0.980)
NPV	0.985 (0.981–0.989)	0.992 (0.989–0.995)	0.997 (0.994–1.000)	0.993 (0.990–0.995)	0.996 (0.993–0.999)
PPV	0.868 (0.819–0.916)	0.938 (0.903–0.974)	0.898 (0.849–0.946)	0.885 (0.847–0.923)	0.974 (0.943–1.000)

In the task of identifying moderate or severe fractures (Table 3B and Fig. 5F), attendings showed the highest AUROC with 0.975, followed by SpineQ (0.968). These two also showed the highest accuracy (> 0.99). Specificity was high for all groups (> 0.99) and sensitivity was highest for attendings (0.956) and SpineQ (0.938). NPV was high with > 0.98 for all groups. PPV was highest for SpineQ with 0.974. Using GLMM for group comparison, SpineQ v1.1 again led in performance, significantly surpassing DL models (*p*_adj_ = 0.0004) and residents (*p*_adj_ = 0.0269). Students performed considerably worse than all other groups, including attendings, residents, SpineQ v1.1 (all *p*_adj_ < 0.0001) and DL models (*p*_adj_ = 0.0023).

Weighted Cohen’s κ showed almost perfect agreement with the reference standard for all groups (> 0.8) except for students. Cohen’s κ for both levels of comparison demonstrated almost perfect agreement for SpineQ (> 0.9), DL models, residents and attendings (all > 0.8, except 0.921 for attendings for differentiation of moderate and severe fractures).

Across upper thoracic, lower thoracic, and lumbar regions, SpineQ v1.1 consistently achieved the highest accuracy and specificity, often outperforming DL models and all reader groups in GLMM comparisons (*p*_adj_ ≤ 0.0001 in most cases). AUROC varied by region, with DL models leading in the upper thoracic, residents and attendings in the lower thoracic and attendings in the lumbar subset for detection of any fracture. SpineQ dominated moderate/severe fracture detection across all subsets, in the lumbar subset comparable to attendings. Students consistently underperformed compared to all other groups.

### Diagnostic performance at the patient level

**Fig. 6:**
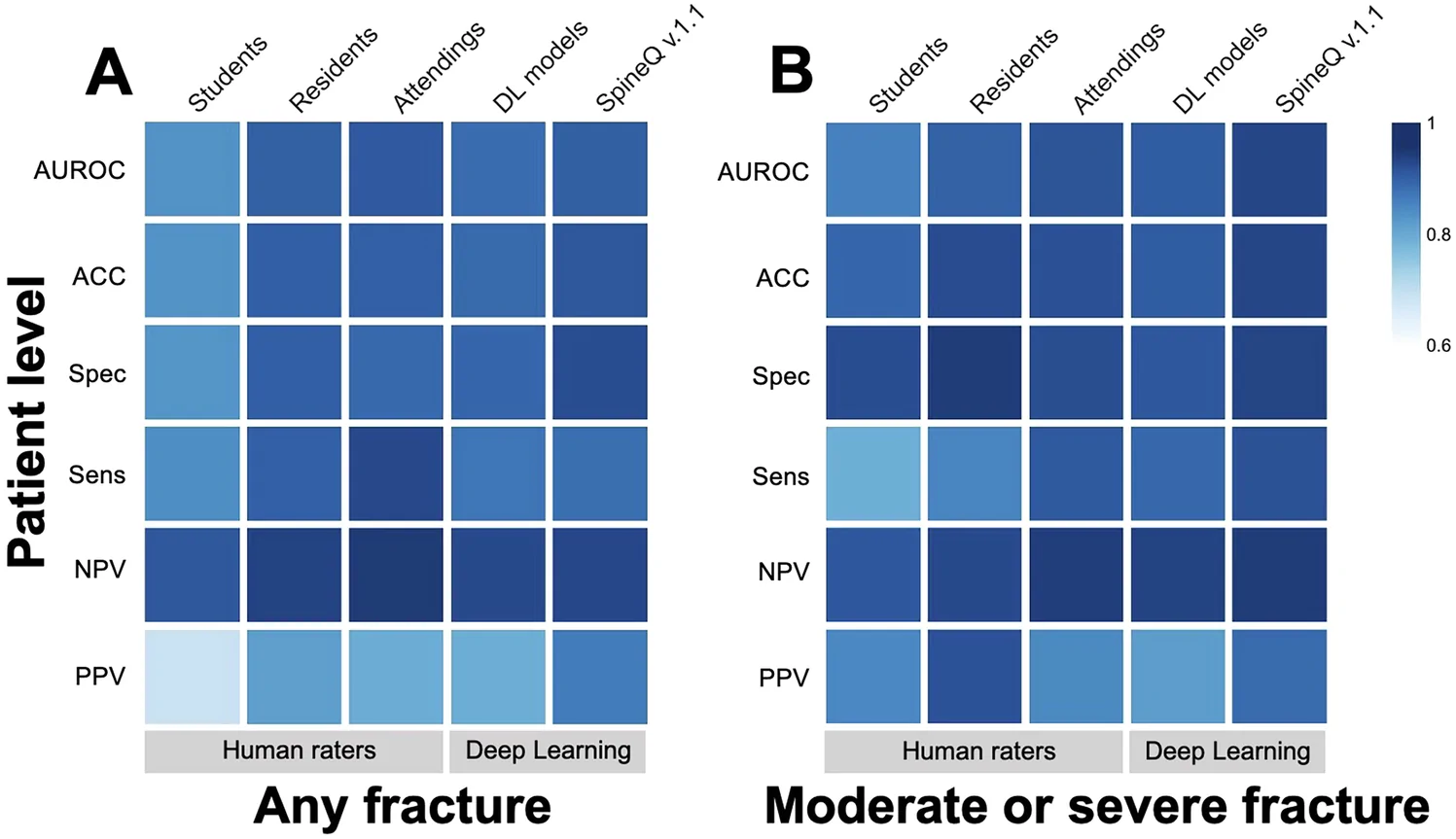
Diagnostic performance on the patient level. Heatmaps representing diagnostic performance for (**A**) discrimination of any fracture (Genant 1–3) from no fracture (Genant 0) and (**B**) differentiation of a “clinically most relevant” moderate or severe fracture (Genant 2 or 3) from no or mild fracture (Genant 0 or 1) on the patient level. ACC, accuracy; NPV, negative predictive value; PPV, positive predictive value; Sens, sensitivity; Spec, Specificity

Performance metrics for detecting any fracture at the patient level are detailed in Table 4A and Fig. 6A. Attendings, residents and SpineQ showed the highest AUROC (≈ 0.94–0.95). SpineQ had the highest Accuracy with 0.952, followed by ≈ 0.94 for residents and attendings. Specificity was with 0.963 highest for SpineQ, whereas sensitivity was highest for attendings with 0.970. Attendings had the highest NPV with 0.990, followed by residents (0.978) and SpineQ (0.971). PPV was highest for SpineQ with 0.897. In statistical group comparisons with GLMM, a trend was observed toward DL models performing worse than SpineQv1.1 (*p*_adj_ = 0.0813). Students performed significantly worse than all other groups (*p*_adj_ < 0.001).

**Table 4:** Diagnostic performance (means of point estimates with 95% confidence intervals calculated using the Wilson methods) for (**A**) discrimination of any fracture (Genant 1–3) from no fracture (Genant 0) and (**B**) differentiation of a “clinically most relevant” moderate or severe fracture (Genant 2 or 3) from no or mild fracture (Genant 0 or 1) on patient level

Metric	Students	Residents	Attendings	DL models	SpineQ v.1.1
**(A) Patient level 0 vs. 123**					
Cohen’s κ	0.676 (0.627–0.725)	0.845 (0.807–0.882)	0.840 (0.795–0.886)	0.808 (0.772–0.843)	0.874 (0.831–0.917)
AUROC	0.865 (0.837–0.893)	0.938 (0.918–0.958)	0.948 (0.928–0.968)	0.919 (0.899–0.939)	0.941 (0.915–0.967)
Accuracy	0.863 (0.841–0.885)	0.939 (0.923–0.954)	0.937 (0.918–0.956)	0.924 (0.909–0.939)	0.952 (0.935–0.969)
Spec	0.860 (0.835–0.886)	0.939 (0.920–0.958)	0.925 (0.900–0.950)	0.929 (0.912–0.946)	0.963 (0.945–0.981)
Sens	0.871 (0.827–0.915)	0.937 (0.904–0.970)	0.970 (0.940–1.000)	0.908 (0.875–0.942)	0.918 (0.873–0.963)
NPV	0.952 (0.934–0.969)	0.978 (0.965–0.990)	0.990 (0.978–1.000)	0.968 (0.955–0.980)	0.971 (0.955–0.987)
PPV	0.701 (0.650–0.752)	0.842 (0.797–0.887)	0.819 (0.763–0.875)	0.818 (0.777–0.859)	0.897 (0.848–0.946)
**(B) Patient level 01 vs. 23**					
Cohen’s κ	0.796 (0.750–0.841)	0.896 (0.862–0.930)	0.882 (0.839–0.925)	0.846 (0.811–0.880)	0.923 (0.888–0.958)
AUROC	0.890 (0.859–0.921)	0.936 (0.911–0.961)	0.954 (0.930–0.977)	0.944 (0.919–0.956)	0.973 (0.948–0.988)
Accuracy	0.930 (0.914–0.947)	0.965 (0.953–0.977)	0.958 (0.942–0.974)	0.944 (0.931–0.957)	0.973 (0.960–0.986)
Spec	0.964 (0.949–0.978)	0.988 (0.979–0.997)	0.962 (0.943–0.980)	0.949 (0.935–0.964)	0.977 (0.963–0.991)
Sens	0.815 (0.762–0.868)	0.883 (0.837–0.929)	0.946 (0.905–0.987)	0.926 (0.892–0.959)	0.959 (0.922–0.996)
NPV	0.949 (0.932–0.965)	0.967 (0.953–0.981)	0.984 (0.972–0.997)	0.978 (0.968–0.988)	0.988 (0.977–0.999)
PPV	0.880 (0.835–0.925)	0.956 (0.924–0.989)	0.878 (0.824–0.932)	0.845 (0.803–0.886)	0.922 (0.875–0.969)

*NPV* negative predictive value, *PPV* positive predictive value, *Sens* sensitivity, *Spec* Specificity

For the task of distinguishing moderate or severe fractures (Genant 2 or 3) from mild or no fractures, results are shown in Table 4B and Fig. 6B. AUROC was highest for SpineQ (0.973) and attendings (0.954), and accuracy was highest for SpineQ and residents with ≈ 0.97. Residents showed the highest specificity with 0.988 and SpineQ the highest sensitivity with 0.959. NPV was highest for SpineQ (0.988) and attendings (0.984). PPV was highest for residents (0.956) and SpineQ (0.922). GLMM was applied to compare group ratings. DL models performed significantly worse than Bonescreen SpineQv1.1 (*p* = 0.004) and residents (*p*_adj_ = 0.037). Students performed significantly worse than attendings (*p*_adj_ = 0.033), residents (*p*_adj_ = 0.001), and SpineQv1.1 (*p*_adj_ < 0.001).

Cohen’s κ showed almost perfect agreement to the reference standard for SpineQ, DL models, attendings and residents (all > 0.8, except 0.923 for SpineQ for differentiation of moderate and severe fractures).

Summed up, at the patient level, SpineQ v1.1 achieved the highest accuracy (0.952) and specificity (0.963) for detecting any fracture, while attendings led in sensitivity (0.970) and NPV (0.990). For moderate/severe fractures, SpineQ showed the best AUROC (0.973) and sensitivity (0.959), with residents leading in specificity (0.988). GLMM comparisons confirmed SpineQ’s significant advantage over DL models (*p* ≤ 0.004) and students, who consistently underperformed.

## Discussion

Our findings demonstrate that SpineQ v1.1 consistently outperformed both deep learning models and human readers across multiple diagnostic tasks and anatomical regions. At the vertebral level, SpineQ achieved the highest diagnostic performance, with significant superiority confirmed by GLMM comparisons (*p*_adj_ ≤ 0.0022). While AUROC for detecting any fracture was comparable between SpineQ, attending radiologists, and residents (≈ 0.93–0.94), SpineQ delivered the highest accuracy and positive predictive value, highlighting its clinical reliability in ‘ruling in’ fractures while effectively eliminating time-consuming false-positive findings. For moderate to severe fractures, SpineQ achieved AUROC and accuracy values exceeding 0.99 and significantly surpassed all other groups (*p*_adj_ ≤ 0.0001). These results underscore SpineQ’s ability to provide reliable and precise fracture detection across the entire spine, outperforming both traditional DL approaches and human expertise in most scenarios. Students consistently demonstrated the lowest diagnostic performance across all comparisons, underscoring that accurate vertebral fracture detection requires substantial training and clinical experience.

Our dataset contained a relatively high proportion of moderate and severe vertebral fractures compared to mild (Genant grade 1) deformities. This reflects the clinical referral context, as patients undergoing CT for pain, suspected fracture, cancer staging, or postoperative assessment are more likely to present with clinically significant deformities [12]. In contrast, epidemiological studies consistently show that mild vertebral fractures are more prevalent in community-dwelling populations, whereas moderate and severe fractures are less frequent [29–31]. While our dataset may not be directly comparable to the general population prevalence of vertebral fracture severity [2], it can be considered representative of real-world CT practice, in which AI models would be applied to help clinicians.

Only a few recent studies have explored the use of deep learning algorithms for detecting thoracic and lumbar vertebral fractures in routine CT scans. Nicolaes et al [32] developed a model trained on abdominal and chest CTs and validated it externally, achieving performance at both the patient and vertebral levels comparable to our student cohort, with particularly high NPV and specificity for moderate to severe fractures. Burns et al [33] introduced a system for detecting, localizing, and classifying compression fractures, reporting sensitivities and accuracies that fell between those of our students and expert radiologists. Glessgen et al [34] applied a deep learning model to the VerSe dataset, achieving accuracy and sensitivity similar to our students, but with higher specificity. Kim et al [35] employed a DenseNet model, reporting an AUC of 0.74 for fracture detection. Bar et al [36] combined a CNN with a recurrent neural network, achieving student-level sensitivity with improved specificity. Tomita et al [37] evaluated various CNN-based approaches for detecting osteoporotic vertebral fractures, matching the performance of an experienced radiologist; however, direct comparison is limited due to the absence of a reference standard. Yilmaz et al [38] used a hierarchical neural network combined with a feed-forward CNN, achieving patient-level sensitivity comparable to residents, attendings, and other machine learning models, albeit with lower specificity. Behanova et al [4] reported an algorithm tested in approximately 200 patients, achieving high accuracy (97%) and specificity (97%), though AUROC was not provided. Page et al [39] found poor performance of a vertebral compression fracture (VCF) algorithm, with sensitivity and specificity for any VCF of 0.66 and 0.90, respectively, and for moderate/severe VCF of 0.78 and 0.87. Bendtsen et al [40] evaluated a commercial algorithm (NANOX), reporting low fracture detection performance (sensitivity 0.68, specificity 0.91).

The comparably higher performance of SpineQ v1.1 on detection and classification accuracy likely stems from its larger training dataset, optimized training procedures, and a refined processing pipeline. The algorithm was specifically trained to detect clinically relevant moderate/severe fractures, while avoiding false positives, aligning with clinical priorities, which is also reflected in generally higher performance in differentiating moderate/severe fractures than any fractures from no fracture. Also, diagnostic accuracy was highest in the lumbar spine and lowest in the upper thoracic region, reflecting both the natural distribution of fractures and the availability of training data.

The consistent high performance of a specially trained algorithm for fracture detection across all evaluation levels underscores the potential of specialized deep learning algorithms trained on large datasets to enhance diagnostic accuracy in vertebral fracture detection. This is particularly relevant given the well-documented variability in human interpretation of vertebral fractures, especially among less experienced raters [12–14], which is also reinforced by the lowest diagnostic performance of medical students across all comparisons in our study. However, even experienced attendings were outperformed by the AI model, suggesting that algorithmic support could be a valuable adjunct in clinical workflows, particularly in high-volume or resource-limited settings. These findings support the growing evidence that deep learning models can match or exceed expert-level performance in medical imaging tasks when trained on large and diverse datasets.

The study has several limitations: The retrospective study design may introduce selection bias and limit causal interpretation. Our cohort was a random collection of hospitalized patients, which may be considered representative of real-world CT practice, but may limit generalizability to screening populations where mild deformities are more common. The predominantly white patient cohort reduces generalizability to more diverse populations. Differences in rater expertise likely influenced the consistency of fracture assessments. Scanner and protocol differences may have affected image quality and fracture assessment and detection but were not included in the analysis. Additionally, our DL models were trained on six fracture categories, while evaluation was performed using only four, which may have affected classification performance.

Future work should extend the reference standard and involve additional senior readers to further strengthen methodological rigor. Expanding cohorts to include more diverse populations and screening settings with mild deformities will improve generalizability. Harmonization of scanner protocols and including scanner type in the analyses, as well as evaluation across all fracture categories, would enhance robustness and clinical applicability of DL models. In addition, subgroup analyses stratified by age, sex, fracture severity, and comorbidities will help to identify clinically relevant performance differences. Finally, integration of DL outputs into radiology workflows and prospective validation in diverse patient cohorts will be critical steps toward bringing these models closer to clinical practice.

## Conclusion

Human experts bring invaluable clinical experience and contextual understanding to medical image interpretation, but their assessments are subject to variability, bias, and increasing workload pressures. Vertebral fractures, as key indicators of osteoporosis, are frequently underdiagnosed in routine CT scans, even by experienced radiologists. In this context, deep learning algorithms offer a promising solution by providing rapid, consistent, and objective assessments. These models can detect subtle patterns and fracture types, including challenging Genant grade 1 deformities, that may easily be overlooked in clinical practice. However, the performance of deep learning models depends heavily on the quality and diversity of training data, and they currently do not incorporate clinical context. Our study demonstrates that a dedicated, commercial algorithm like SpineQ, trained explicitly for vertebral fracture detection, can compare to expert-level performance and highlights that deep learning tools hold strong potential to support clinicians in improving correct diagnosis and reducing missed fractures, ultimately enhancing patient care.

## Data Availability

Training data remains hidden due to ethical and proprietary constraints; test data stems from the publicly available VerSe 19 & 20 datasets [19–21]. Reference standard readings generated for lumbar and thoracic vertebrae have been deposited in the Open Science Framework (OSF) and can be accessed at https://osf.io/4skx2/files/zy68u.

## Abbreviations

AI: Artificial intelligence
AUROC: Area under the curve
CNNs: Convolutional neural networks
GLMMs: Generalized linear mixed models
NPV: Negative predictive value
PPV: Positive predictive value
Sens: Sensitivity
Spec: Specificity
VCF: Vertebral compression fracture
VerSe: Vertebral segmentation
