# Correction: Diagnostic accuracy of deep learning vs. human raters for detecting osteoporotic vertebral compression fractures in routine CT scans

**DOI:** 10.1007/s00330-026-12637-x

**Published:** 2026-08-06

**Authors:** Evamaria O. Riedel, David Schinz, Matthias Keicher, Sebastian Rühling, Malek El Husseini, Chantal Pellegrini, Thomas Baum, Michael Dieckmeyer, Luca Malagutti, Isabel Seeger, Anna S. Walburga, Benedikt Wiestler, Nico Sollmann, Maximilian T. Löffler, Arthur Wagner, Jan S. Kirschke

**Affiliations:** 1 Department of Neuroradiology, School of Medicine and Health, Technical University of Munich, Munich, Germany; 2 Institute of Radiology, Universitätsklinikum Erlangen, Friedrich-Alexander-Universität Erlangen-Nürnberg, Erlangen, Germany; 3 Computer-Aided Medical Procedures, Technical University of Munich, Munich, Germany; 4 Department of Diagnostic, Interventional, and Pediatric Radiology, Inselspital, University of Bern, Bern, Switzerland; 5 TranslaTUM-Central Institute for Translational Cancer Research, Technical University of Munich, Munich, Germany; 6 AI for Image-Guided Diagnosis and Therapy, School of Medicine and Health, Technical University of Munich, Munich, Germany; 7 Department of Diagnostic and Interventional Radiology, University Hospital Ulm, Ulm, Germany; 8 Department of Nuclear Medicine, University Hospital Ulm, Ulm, Germany; 9 TUM-Neuroimaging Center, TUM Klinikum Rechts der Isar, Technical University of Munich, Munich, Germany; 10 Department of Diagnostic and Interventional Radiology, University Medical Center Freiburg, Freiburg, Germany; 11 Department of Neurosurgery, University Hospital, Technical University Munich School of Medicine, Munich, Germany

**Correction to: European Radiology**

https://doi.org/10.1007/s00330-026-12393-y, published online 24 February 2026

The authors regret to inform that an error occurred in the statistical analysis presented in Tables 1–4 of the original article. Due to a default setting in the statistical software (R), the negative class (no fracture) was incorrectly assigned as the ‘positive class’ for the calculation of sensitivity, specificity, positive predictive value (PPV), and negative predictive value (NPV).

Specifically, the values labeled as ‘Sensitivity’ in the original tables represent the ‘Specificity’ (detection of non-fractured vertebrae), and vice versa. Similarly, PPV and NPV values were transposed. This systematic labeling error affects all sub-analyses (in Tables 1–4, as well as the corresponding Figures 3, 5, and 6, and the descriptions in the “Results” and the first paragraph of the “Discussion” sections).

The authors emphasize that the Kappa, Accuracy and AUROC values, as well as the overall ranking of the models and human raters, remain unaffected by this labeling error. The core conclusion—that specifically trained deep learning models achieve diagnostic performance comparable to or exceeding that of human experts—remains valid.

To correct this, the labels “Spec” & “Sens” and “NPV” & “PPV” were transposed in the graphical abstract, in Figures 3, 5 and 6, and in the first column of tables 1, 2, 3 and 4.

Furthermore, the following changes were made to the text:

## Diagnostic performance at the vertebral level

In the first paragraph, a sentence incorrectly stated “SpineQ and DL models were more sensitive, and human raters were more specific. PPV was comparably high for all groups (< 0.99). NPV was highest for SpineQ (0.962) and DL models (0.894) and lower for all human raters.” and was corrected to “SpineQ and DL models were more specific, and human raters were more sensitive. NPV was comparably high for all groups (< 0.99). PPV was highest for SpineQ (0.962) and DL models (0.894) and lower for all human raters.”

In the second paragraph, a sentence incorrectly stated “While sensitivity was comparably high for all groups (> 0.99), specificity was highest for SpineQ (0.929) and attendings (0.904). PPV was > 0.99 for all groups except for students, and NPV was highest for SpineQ (0.970).” and was corrected to “While specificity was comparably high for all groups (> 0.99), sensitivity was highest for SpineQ (0.929) and attendings (0.904). NPV was > 0.99 for all groups except for students, and PPV was highest for SpineQ (0.970).

In the fourth paragraph, a sentence incorrectly stated “For detecting any fracture, AUROC was comparable for SpineQ, attendings, and residents (≈0.93–0.94), but lower for DL models and students; accuracy and NPV were highest for SpineQ and DL models, while human raters were more specific.” and was corrected to “For detecting any fracture, AUROC was comparable for SpineQ, attendings, and residents (≈0.93–0.94), but lower for DL models and students; accuracy and PPV were highest for SpineQ and DL models, while human raters were more sensitive.”.

## Diagnostic performance at the vertebral level for upper, lower thoracic, and lumbar subsets

### Upper thoracic vertebrae

In the first paragraph, a sentence incorrectly stated “SpineQ showed the highest sensitivity with 1.00, and DL models showed the highest specificity with 0.86. PPV was high for all groups (> 0.98) while NPV was considerably highest for SpineQ with 1.00 (all other groups < 0.80).” and was corrected to “SpineQ showed the highest specificity with 1.00, and DL models showed the highest sensitivity with 0.86. NPV was high for all groups (> 0.98) while PPV was considerably highest for SpineQ with 1.00 (all other groups < 0.80).”

In the second paragraph, a sentence incorrectly stated “All groups were highly sensitive (*p* > 0.99), with the highest specificity for SpineQ (0.875). PPV was high for all groups (> 0.99), and NPV was higher for all human raters than for DL models and SpineQ.” and was corrected to “All groups were highly specific (*p* > 0.99), with the highest sensitivity for SpineQ (0.875). NPV was high for all groups (> 0.99), and PPV was higher for all human raters than for DL models and SpineQ.”

### Lower thoracic vertebrae

In the first paragraph, a sentence incorrectly stated “SpineQ and DL models were most sensitive with values > 0.99, and residents and attendings were most specific (> 0.90). PPV was high for all groups (> 0.99), and NPV was highest for SpineQ (0.912) and DL models (0.84).” and was corrected to “SpineQ and DL models were most specific with values > 0.99, and residents and attendings were most sensitive (0.90). NPV was high for all groups (> 0.99), and PPV was highest for SpineQ (0.912) and DL models (0.84).”

In the second paragraph, a sentence incorrectly stated “Accuracy was high with values > 0.99 for SpineQ, residents and attendings and > 0.98 for ML models and students. Sensitivity and specificity were both highest for SpineQ (1.000 and 0.932, respectively). PPV was highest for SpineQ with 0.998 and values > 0.98 for all groups. NPV was 1.00 for SpineQ.” and was corrected to “Accuracy was high with values > 0.99 for SpineQ, residents and attendings and > 0.98 for DL models and students. Specificity and sensitivity were both highest for SpineQ (1.000 and 0.932, respectively). NPV was highest for SpineQ with 0.998 and values > 0.98 for all groups. PPV was with 1.00 highest for SpineQ.”

### Lumbar vertebrae

In the first paragraph, a sentence incorrectly stated “Sensitivity was highest for SpineQ and DL models (0.999 and 0.994, respectively). Specificity was higher for all human rater groups than for SpineQ and DL models. PPV was highest for attendings (0.998) with high performances for all groups (> 0.99, except DL models with 0.983). NPV was highest for SpineQ (0.989), followed by DL models (0.971).” and was corrected to “Specificity was highest for SpineQ and DL models (0.999 and 0.994, respectively). Sensitivity was higher for all human rater groups than for SpineQ and DL models. NPV was highest for attendings (0.998) with high performances for all groups (> 0.99, except DL models with 0.983). PPV was highest for SpineQ (0.989), followed by DL models (0.971).”

In the second paragraph, a sentence incorrectly stated “Sensitivity was high for all groups (> 0.99), and specificity was highest for attendings (0.956) and SpineQ (0.938). PPV was high with > 0.98 for all groups. NPV was highest for SpineQ with 0.974.” and was corrected to “Specificity was high for all groups (> 0.99), and sensitivity was highest for attendings (0.956) and SpineQ (0.938). NPV was high with > 0.98 for all groups. PPV was highest for SpineQ with 0.974.”.

In the fourth paragraph, a sentence incorrectly stated “Across upper thoracic, lower thoracic, and lumbar regions, SpineQ v1.1 consistently achieved the highest accuracy and sensitivity, …” and was corrected to “Across upper thoracic, lower thoracic, and lumbar regions, SpineQ v1.1 consistently achieved the highest accuracy and specificity, …”.

## Diagnostic performance at the patient level

In the first paragraph, a sentence incorrectly stated “Sensitivity was with 0.963 highest for SpineQ, whereas specificity was highest for attendings with 0.970. Attendings had the highest PPV with 0.990, followed by residents (0.978) and SpineQ (0.971). NPV was highest for SpineQ with 0.897.” and was corrected to: “Specificity was with 0.963 highest for SpineQ, whereas sensitivity was highest for attendings with 0.970. Attendings had the highest NPV with 0.990, followed by residents (0.978) and SpineQ (0.971). PPV was highest for SpineQ with 0.897.”

In the second paragraph, a sentence incorrectly stated “Residents showed the highest sensitivity with 0.988, and SpineQ showed the highest specificity with 0.959. PPV was highest for SpineQ (0.988) and attendings (0.984). NPV was highest for residents (0.956) and SpineQ (0.922).” and was corrected to “Residents showed the highest specificity with 0.988, and SpineQ showed the highest sensitivity with 0.959. NPV was highest for SpineQ (0.988) and attendings (0.984). PPV was highest for residents (0.956) and SpineQ (0.922).”

In the fourth paragraph, a sentence incorrectly stated “Summed up, at the patient level, SpineQ v1.1 achieved the highest accuracy (0.952) and sensitivity (0.963) for detecting any fracture, while attendings led in specificity (0.970) and PPV (0.990). For moderate/severe fractures, SpineQ showed the best AUROC (0.973) and specificity (0.959), with residents leading in sensitivity (0.988).” and was corrected to “Summed up, at the patient level, SpineQ v1.1 achieved the highest accuracy (0.952) and specificity (0.963) for detecting any fracture, while attendings led in sensitivity (0.970) and NPV (0.990). For moderate/severe fractures, SpineQ showed the best AUROC (0.973) and sensitivity (0.959), with residents leading in specificity (0.988).”

**Fig. 4:**
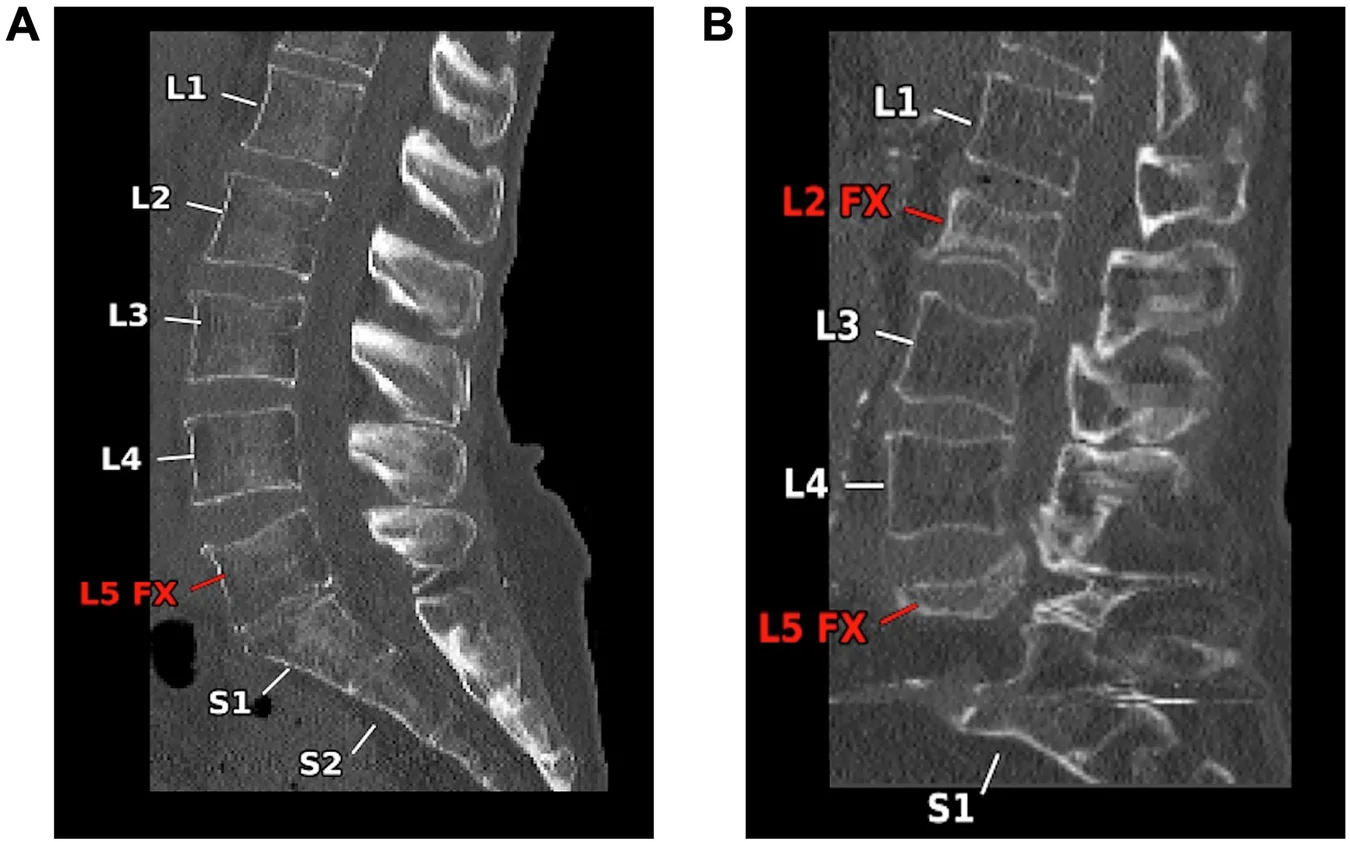
Examples of incorrectly identified fractures. **A** A grade 1 fracture, which was overlooked by two of the three students and three of the four deep learning (DL) models. One DL model and one attending physician incorrectly classified it as a Genant grade 2 fracture. **B** Genant grade 3 fractures in L2 and L5 were misclassified as grade 2 by one attending, all residents, and two students. Additionally, the L5 fracture was not detected by any of the DL models. FX, fracture

## Discussion

In the first paragraph, a sentence incorrectly stated “…, SpineQ delivered the highest accuracy and negative predictive value, highlighting its robustness in ruling out disease,” and was corrected to “…, SpineQ delivered the highest accuracy and positive predictive value, highlighting its clinical reliability in ‘ruling in’ fractures while effectively eliminating time-consuming false-positive findings.”

In addition, Figure 4 has been updated in the original publication to resolve a formatting and cropping error at the L5/S1 level in Panel B and in the corresponding Legend FX, fracture has been replaced with with G = Genant grade.

For completeness and transparency, the old incorrect version is displayed below.

The original article has been corrected.

